# Genomic Prediction Accuracy of Stripe Rust in Six Spring Wheat Populations by Modeling Genotype by Environment Interaction

**DOI:** 10.3390/plants11131736

**Published:** 2022-06-30

**Authors:** Kassa Semagn, Muhammad Iqbal, Diego Jarquin, Harpinder Randhawa, Reem Aboukhaddour, Reka Howard, Izabela Ciechanowska, Momna Farzand, Raman Dhariwal, Colin W. Hiebert, Amidou N’Diaye, Curtis Pozniak, Dean Spaner

**Affiliations:** 1 Department of Agricultural, Food and Nutritional Science, 4-10 Agriculture-Forestry Centre, University of Alberta, Edmonton, AB T6G 2P5, Canada; mi1@ualberta.ca (M.I.); izabela@ualberta.ca (I.C.); farzand@ualberta.ca (M.F.); 2 Agronomy Department, University of Florida, Gainesville, FL 32611, USA; jhernandezjarqui@ufl.edu; 3 Lethbridge Research and Development Centre, Agriculture and Agri-Food Canada, 5403-1st Avenue South, Lethbridge, AB T1J 4B1, Canada; harpinder.randhawa@agr.gc.ca (H.R.); reem.aboukhaddour@canada.ca (R.A.); raman.dhariwal@agr.gc.ca (R.D.); 4 Department of Statistics, University of Nebraska–Lincoln, Lincoln, NE 68583, USA; rekahoward@unl.edu; 5 Morden Research and Development Centre, Agriculture and Agri-Food Canada, 101 Route 100, Morden, MB R6M 1Y5, Canada; colin.hiebert@agr.gc.ca; 6 Crop Development Centre and Department of Plant Sciences, University of Saskatchewan, 51 Campus Drive, Saskatoon, SK S7N 5A8, Canada; amidou.ndiaye@usask.ca (A.N.); curtis.pozniak@usask.ca (C.P.)

**Keywords:** disease resistance, genome-wide selection, G × E interaction, prairie provinces, prediction accuracy, priority disease, Western Canada

## Abstract

Some previous studies have assessed the predictive ability of genome-wide selection on stripe (yellow) rust resistance in wheat, but the effect of genotype by environment interaction (GEI) in prediction accuracies has not been well studied in diverse genetic backgrounds. Here, we compared the predictive ability of a model based on phenotypic data only (M1), the main effect of phenotype and molecular markers (M2), and a model that incorporated GEI (M3) using three cross-validations (CV1, CV2, and CV0) scenarios of interest to breeders in six spring wheat populations. Each population was evaluated at three to eight field nurseries and genotyped with either the DArTseq technology or the wheat 90K single nucleotide polymorphism arrays, of which a subset of 1,058- 23,795 polymorphic markers were used for the analyses. In the CV1 scenario, the mean prediction accuracies of the M1, M2, and M3 models across the six populations varied from −0.11 to −0.07, from 0.22 to 0.49, and from 0.19 to 0.48, respectively. Mean accuracies obtained using the M3 model in the CV1 scenario were significantly greater than the M2 model in two populations, the same in three populations, and smaller in one population. In both the CV2 and CV0 scenarios, the mean prediction accuracies of the three models varied from 0.53 to 0.84 and were not significantly different in all populations, except the Attila/CDC Go in the CV2, where the M3 model gave greater accuracy than both the M1 and M2 models. Overall, the M3 model increased prediction accuracies in some populations by up to 12.4% and decreased accuracy in others by up to 17.4%, demonstrating inconsistent results among genetic backgrounds that require considering each population separately. This is the first comprehensive genome-wide prediction study that investigated details of the effect of GEI on stripe rust resistance across diverse spring wheat populations.

## 1. Introduction

Stripe rust is one of the most devastating fungal diseases reported in wheat-growing countries globally, which is caused by *Puccinia* *striiformis* f. sp. *tritici* (*Pst*). *Pst* is an airborne pathogen that can travel thousands of miles and cause stripe rust epidemics, primarily at higher elevations, humid conditions, and cooler climates with 7–12 °C nighttime temperatures and 20–26 °C daytime temperatures [1]. However, the pathogen has gradually expanded into warmer regions in all continents except Antarctica [2]. *Pst* has been estimated to infect about 88% of the world’s wheat production, causing an annual loss of 5.5 million tons of wheat grains worth USD 979 million [3]. The disease symptoms include elongated lesions bearing yellow-orange streaks (pre-pustules), followed by small, bright yellow, elongated uredial pustules arranged in conspicuous rows on the leaves. The disease can cause up to 100% grain yield and quality losses (often 40–70%) depending on the genetics of the varieties, the stage of plant development at the time of infection, the suitability of the weather conditions for *Pst* development, and the virulence of the *Pst* isolates [1,4,5]. Although all plant growth stages are susceptible to infection, high levels of stripe rust infestation before or during heading usually have the greatest effect on grain yield. *Pst* is also more severe on late-maturing than early-maturing cultivars.

In Canada, wheat is a major crop with an estimated total production of 35.2 million tons in 2020, of which the three prairie provinces of Manitoba, Alberta, and Saskatchewan accounted for over 90% of the Canadian wheat production (https://www.statcan.gc.ca (accessed on 28 June 2022)). Of the nine milling classes grown in the prairie provinces, *Pst* epidemics have been reported in the Canada western soft white wheat (CWSWS), Canada Western Red Spring (CWRS), Canada Prairie Spring Red (CPSR), Canada Prairie Spring White (CPSW), Canada Western Hard White Spring (CWHWS), and Canada Western Red Winter (CWRW) [4,6]. Alberta is the stripe rust hotspot province due to its proximity to the Pacific Northwestern United States, which has been reported as having unusually high stripe rust epidemics since the 1970s [7]. The predominant stripe rust races and isolates identified in Western Canada primarily originated from the Pacific Northwestern USA [8,9]. In recent years, *Pst* has been regularly found in Western Canada, which requires spraying fungicides to minimize the disease. The stripe rust pathogen is also highly aggressive due to rapid changes in its population structure and virulence [9]. As a result, stripe rust is one of the five priority diseases with at least an intermediate level of resistance required for variety registration and release in the three prairie provinces [4].

Of the different methods employed to minimize the impact of the *Pst* pathogen, the development and cultivation of disease-resistant varieties is an environmentally safe, durable, and cost effective approach to maintaining yield stability and quality [10]. Resistance breeding requires searching for suitable resistance donor parents and transferring the new source(s) of resistance into an elite genetic background using conventional and modern breeding methods, including genome-wide (genomic) selection. Over 83 race-specific *Yr* genes [11] and 384 quantitative trait loci (http://www.wheatqtldb.net/fungal_new.php (accessed on 28 June 2022)) that control adult plant stripe rust resistance have been reported on all wheat chromosomes [12]. Of the 83 *Yr* resistance genes that have been reported in wheat and its relatives [11], only a few genes (*Yr1*, *Yr5*, *Yr15*, *Yr17*, *Yr18/Lr34*, *Yr29*, *Yr36,* and *Yr76*) are still effective against the prevalent *Pst* races in Canada [4,6,9]. Unfortunately, resistance due to single genes can lose its effectiveness over time due to changes in pathogen populations. Most Canadian breeders focus mainly on *Yr18*, and somewhat on *Yr17* and *Yr36* [4,13]. The need in combining multiple resistance genes and major effect QTLs into “pyramids” using marker-assisted selection (MAS) has been frequently cited to increase the level and durability of resistance. However, there are still issues that are not yet well understood, including the actual number of genes and QTLs that can be stacked in each pyramid, how stacked resistance genes and QTLs express across different genetic backgrounds, and how the resistance gene and QTL combinations interact with each other [14].

In contrast to MAS that requires flanking, reproducible, and breeder-friendly molecular markers linked with target genes and major effect QTLs, genomic selection (GS) uses all genome-wide molecular markers to predict the most likely phenotypic performance of (i) a subset of lines that have not been phenotyped in any environment (newly developed lines, CV1), (ii) a subset of lines that have been evaluated in some environments but not in other environments (a sparse testing, CV2), and/or (iii) lines that have been evaluated in some environments but are completely missing at other environments (predicting an entire environment, CV0) [15,16]. Previous proofs of concept GS studies conducted on stripe rust resistance in wheat reported inconsistent prediction accuracies ranging from 0.12 to 0.79, depending on the models, cross-validations (CV) scenarios, and genetic backgrounds [17,18,19,20,21,22,23,24]. Except for our recent study that investigated the potential of GS on Fusarium head blight (FHB), common bunt, leaf spot, leaf rust, and stripe rust [22], all other previous studies did not consider the inclusion of the genotype by environment interaction (GEI) via the multiplicative reaction norm models [25]. The reaction norm model that incorporates GEI (M3) involves partitioning variance components into environments (E), genotypes (L), the main effect of the molecular markers or genomics (G), and GEI, which generally improves the predictive ability over the main effect of the M2 model. As compared with the main effect M2 model, the M3 model increased prediction accuracies of the five major wheat diseases on average by 6.1% in CV1, 3.2% in CV2, and 1.6% in CV0. Leaf spot recorded in two of the three populations showed up to 53.9% greater accuracies in the M3 than in the M2 model [22]. For stripe rust, the M3 model increased prediction accuracies across three spring wheat populations by 0.3–2.2% in CV0, 0.8–14.6% in CV1, and 1.2–4.6% in CV2. Here, we extended our previous study by adding more data to compare (1) variance components and broad-sense heritability, and (2) the prediction accuracies of the three models and CV scenarios in six diverse spring wheat populations that represented an association mapping (diversity) panel of historical and modern varieties, doubled haploid (DH) lines, and recombinant inbred lines (RILs).

## 2. Results

### 2.1. Phenotypic Variation and Variance Components

**Figure 1:**
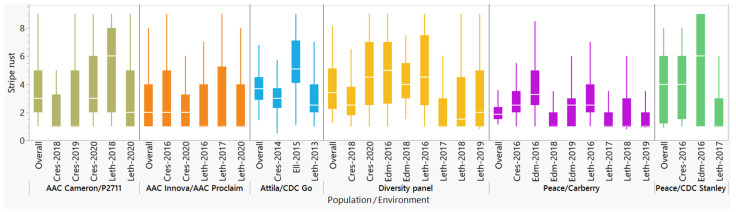
Comparison of reaction to stripe rust recorded in six spring wheat populations evaluated across all three to eight field environments (overall) and each environment. The individual environment starts with a prefix representing location (Cres: Creston, Leth: Lethbridge, Edm: Edmonton, Ell: Ellerslie), followed by the year of the trial.

**Figure 2:**
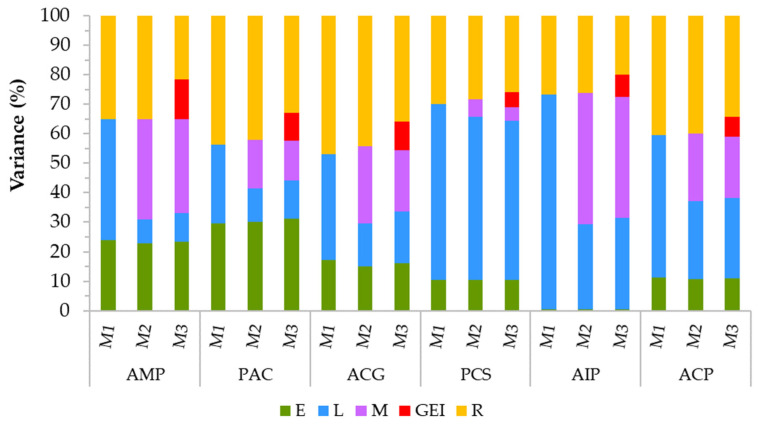
Partitioning of total variance components into environments (E), genotypes (L), molecular markers (M), interactions between genotypes and environment (GEI), and residual (R) components using the baseline M1 model, the main effect reaction norm M2 model, and the M3 model that incorporated GEI. Population codes are as follows: AMP (diversity panel), PAC (Peace/Carberry), ACG (Attila/CDC Go), PCS (Peace/CDC Stanley), AIP (AAC Innova/AAC Proclaim), and ACP (AAC Cameron/P2711).

Stripe rust scores of the six populations were highly variable depending on the genetic backgrounds and the environments (Figure 1). In the combined data of all environments, genotypes (lines) and GEI showed highly significant differences (*p* < 0.01) in all populations (Table 1). Environmental variances were significant in four populations (the diversity panel, Peace/Carberry, Peace/CDC Stanley, and AAC Cameron/P2711) but not in the Attila/CDC Go and AAC Innova/AAC Proclaim populations. Broad-sense heritability estimated from all environments varied from 0.62 in the Attila/CDC Go to 0.94 in the AAC Innova/AAC Proclaim populations. Figure 2 summarizes the proportion of variance components due to environments, genotypes, molecular markers, residual, and/or GEI components using the three models. In all three models, environmental, genotype, and residual variances varied from 0.4 to 31.2%, from 8.1 to 72.7%, and from 19.8 to 47.0%, respectively. Molecular markers were used in both the M2 and M3 models, which accounted for 4.7–44.4% of the variances. GEI was estimated only in the M3 model, which varied from 5.2% in the Peace/CDC Stanley to 13.5% in the diversity panel. When variance components computed only with the M3 model were considered, environments, genotypes, molecular markers, GEI, and residual variances accounted for an average of 15.4%, 25.4%, 22.1%, 8.7%, and 28.3% per population, respectively. The lowest and highest environmental variances in the M3 model were observed in the AAC Innova/AAC Proclaim and Peace/Carberry populations, respectively (Figure 2).

### 2.2. Comparisons of Prediction Models Based on CV1

**Figure 3:**
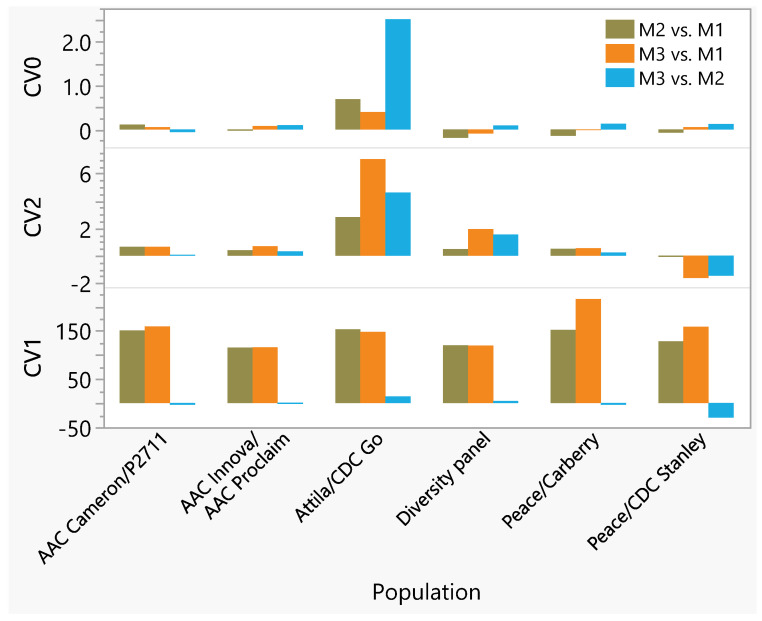
Comparisons of overall average changes in prediction accuracies of stripe rust resistance between two models in six spring wheat populations evaluated at three to eight environments. For each cross-validation (CV) scenario, the changes were computed as follows: M2 vs. M1 =$\;\frac{\;100\left(M2-M1\right)}{\begin{array}{c} M2 \end{array}}$; M3 vs. M1 =$\;\frac{100\left(M3-M1\right)}{M3}$, and M3 vs. M2 = $\frac{100\left(M3-M2\right)}{M3}$.

Three-dimensional plots of PC1 (which accounted for 4.6–11.3% of the genetic variation depending on the population), PC2 (4.2–7.6%), and PC3 (3.7–6.6%) revealed no clear population structure within and among populations (Figure S1). The mean prediction accuracies of the M1 model with the CV1 scenario across the six populations were negative or near zero, which varied from −0.11 to −0.07 (Table 2). The M2 model increased prediction accuracies of presumably newly developed lines between 0.22 and 0.49 (overall mean = 0.31), which was 114.1–144.2% (mean 133.4%) greater than the M1 model (Table S1), suggesting the major effect of molecular markers in increasing prediction accuracies over the disease score phenotype alone. Similarly, the mean prediction accuracies of the M3 model in presumably newly developed lines varied between 0.19 and 0.48 (overall mean = 0.32), which was 114.2–146.9% (mean 133.7%) greater than the M1 model. ANOVA with the Tukey–Kramer HSD test revealed significantly smaller prediction accuracies in the M1 than both the M2 and M3 models regardless of the populations (Table S2A). As compared with the M2 model, the Tukey–Kramer HSD test revealed significantly greater accuracies in the M3 model in both the Attila/CDC Go population and the diversity panel, with the same accuracies in three populations (AAC Cameron/P2711, AAC Innova/AAC Proclaim, and Peace/Carberry) and smaller accuracies in the Peace/CDC Stanley population. In five of the six populations, therefore, the M3 model incorporating the GEI in the CV1 scenario provided either similar or significantly greater accuracies over the main effect M2 model in predicting the performance of a subset of newly developed lines not tested in any environment. The M3 model improved the overall prediction accuracies over the M2 model in the CV1 by 4.3% in the diversity panel, by 5.6% in the Peace/Carberry, and by 12.4% in the Attila/CDC Go populations, but it decreased accuracies by 0.5% in the AAC Innova/AAC Proclaim, by 5.5% in the AAC Cameron/P27, and by 17.4% in the Peace/CDC Stanley populations (Figure 3, Table S1B). Such results demonstrate the inconsistency in the effect of incorporating GEI in the prediction model, with some populations showing advantages and others a disadvantage.

### 2.3. Comparisons of Models Based on CV2 and CV0

In the CV2 scenario, the mean prediction accuracies of a subset of lines that have been tested in some environments but not in others ranged from 0.53 to 0.82 in the M1, from 0.54 to 0.82 in the M2, and from 0.57 to 0.83 in the M3 models. In the CV0 scenario, the mean accuracies of all lines that have been tested in some environments but missing in others ranged from 0.54 to 0.84 in the M1, from 0.53 to 0.84 in the M2, and from 0.54 to 0.84 in the M3 models (Table 2). ANOVA performed on prediction accuracies of the CV2 and CV0 scenarios revealed no significant differences in prediction accuracies among the three models in all populations except the Attila/CDC Go where the M3 model in the CV2 scenario gave greater accuracy than both the M1 and M2 models (Table S2). The M3 model increased the overall mean prediction accuracies over the M2 model in the CV2 scenario by 0–7.1% (mean 1.4%) in all populations except the Peace/CDC Stanley, which showed a 1.6% smaller accuracy (Figure 3, Table S1B). In the CV0 scenario, all the three models provided the same predictions in all six populations and the changes in the overall mean prediction accuracies between the M3 and M2 models were nearly zero.

### 2.4. Prediction Accuracies of the M3 Model across Populations and Environments

**Figure 4:**
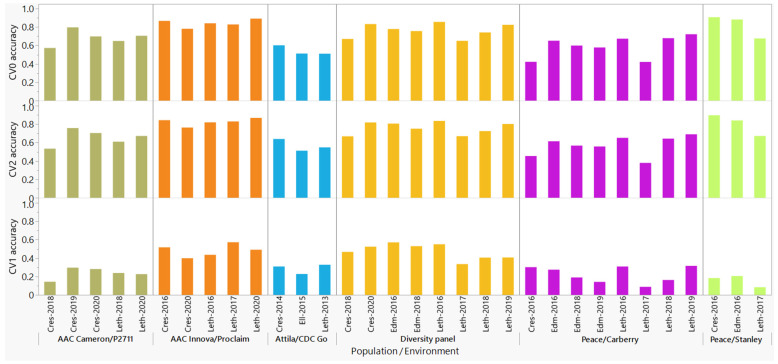
Comparisons of prediction accuracies of stripe rust resistance in six spring wheat populations evaluated at three to eight environments using the M3 model and three random cross-validation scenarios (CV0, CV1, and CV2). In each population, CV1, CV2, and CV0 represent predicting the performance of a subset of 25% of newly developed lines that have not been tested in any environment, a subset of 25% of lines tested in some but not other environments (sparse testing), and all lines tested in some environments but missing in others, respectively. Each environment starts with a prefix representing the experimental site (Cres: Creston, Leth: Lethbridge, Edm: Edmonton, Ell: Ellerslie) followed by the year of the trial.

Stripe rust prediction accuracies in both the CV2 and CV0 scenarios were significantly greater than in the CV1 scenario regardless of the models and populations (Table S2B). In each CV scenario, we then compared the predictive ability of the M3 model among environments to understand if some environments provided greater accuracies than others (Figure 4, Table S3A). In the CV1 scenario, the mean prediction accuracies per environment varied from 0.33 to 0.57 in the diversity panel, from 0.14 to 0.32 in the Peace/Carberry, from 0.23 to 0.33 in the Attila/CDC Go, from 0.17 to 0.21 in the Peace/CDC Stanley populations, from 0.40 to 0.57 in the AAC Innova/AAC Proclaim, and from 0.14 to 0.30 in the AAC Cameron/P2711. In each population, the highest prediction accuracies in the CV1 scenario were observed at the University of Alberta South Campus in Edmonton in 2016 (0.57) in the diversity panel, at the Lethbridge Research and Development Center (Lethbridge RDC) in Alberta in 2019 (0.32) in the Peace/Carberry, at Lethbridge RDC in 2013 (0.33) in the Attila/CDC Go population, at the University of Alberta South Campus in Edmonton in 2016 (0.21) in the Peace/CDC Stanley, at the Lethbridge RDC in 2017 (0.57) in the AAC Innova/AAC Proclaim, and near Creston in British Columbia in 2019 (0.30) in the AAC Cameron/P2711 populations. In both CV2 and CV0 scenarios, prediction accuracies computed for individual environments in each population ranged from 0.38 to 0.91. The highest accuracy was observed at the Lethbridge RDC in 2016 (0.84–0.86) in the diversity panel, at the Lethbridge RDC in 2019 (0.69–0.72) in the Peace/Carberry population, at Creston station in 2014 (0.60–0.64) in the Attila/CDC Go population, at Creston station in 2016 (0.90–0.91) in the Peace/CDC Stanley population, at the Lethbridge RDC in 2020 (0.87–0.89) in the AAC Innova/AAC Proclaim population, and at the Creston station in 2019 (0.76–0.80) in the AAC Cameron/P2711 populations. Although a few environments provided similar prediction accuracies, most environments differed significantly in prediction accuracies regardless of the CV scenarios (Table S3A).

We also compared the mean prediction accuracies of the six populations using ANOVA and the Tukey–Kramer HSD comparisons of means irrespective of the environments, which revealed significantly greater prediction accuracies in both the AAC Innova/AAC Proclaim and the diversity panel in the CV1 (0.47–0.48), CV2 (0.80–0.83), and CV0 (0.82–0.84) than all the other four populations (Table S3B). The Peace/CDC Stanley population in the CV1 (0.16), both Peace/Carberry and the Attila/CDC Go in the CV2 (0.57), and Attila/CDC Go in the CV0 (0.54) had the lowest prediction accuracies among all other populations.

## 3. Discussion

Multiple studies have shown that integrating GS in breeding programs reduces breeding cycle duration, increases the selection accuracy, accelerates the rate of genetic gains per unit time and cost, and/or reduces costs of large-scale multi-environment phenotyping of breeding lines [26,27,28,29,30]. For such purposes, GS facilitates the development of improved germplasm by predicting the performance of lines using three scenarios that plant breeders have widely used. CV1 is one of the scenarios used to predict the most likely performance of a subset of lines that have not yet been evaluated in any environment but genotyped with genome-wide markers [31]. In the present study, the stripe rust prediction accuracies in the CV1 were significantly smaller than both the CV2 and CV0 regardless of the models and populations, which were negative or nearly zero when only phenotypes were used in the M1 model and low to moderate (0.19–0.49) when both phenotypes and molecular markers were used with and without incorporating GEI (Table 2, Table S2B). Such results restrict the breeder’s ability to confidently implement GS to develop stripe-rust-resistant spring wheat varieties, which agree with several previous studies that reported similar accuracies in multiple traits [22,32,33,34,35,36].

The most encouraging results across the six spring wheat populations were observed in both the CV2 (sparse testing) and CV0 (predicting new environments) regardless of the models and populations. In all three models, the mean stripe rust prediction accuracies of the six Canadian spring wheat populations in the CV2 and CV0 scenarios varied from 0.54 to 0.84 (Table 2), which was greater than several other studies conducted on wheat. Previous GS studies conducted in non-Canadian wheat germplasm reported highly variable accuracies, which ranged from 0.39 to 0.56 in tetraploid wheat [37], from 0.16 to 0.72 in winter wheat [18,19,20,38], and from 0.12 to 0.79 in diverse spring wheat populations [17,18,21,39,40]. Overall, our results provided a strong justification for wheat breeders for integrating GS in developing stripe rust resistance spring wheat germplasm using the CV2 and/or CV0 scenarios regardless of the models and genetic background, which would reduce the cost, resources, and logistics associated with field evaluation by at least 25%. In addition, GS could significantly reduce the time required for developing and registering/releasing improved varieties [27,41].

Several studies have reported inconsistencies in the presence and magnitude of GEI depending on populations and traits for multiple reasons, including selection history in the germplasm, genetic drift, and environmental variation [42], which was evident in the present study (Figure 2). Statistically significant differences in prediction accuracies between the M2 and M3 models were mainly observed in the CV1 but not in the CV2 or CV0 scenario (Table S2A). In three of the six populations (diversity panel, Attila/CDC GO, and Peace/Carberry), GEI accounted for 9.5–13.2% of the total variation (Figure 2), with the M3 model showing 4.3–12.4% greater prediction accuracies over the main effect M2 model (Table S1B). In the Peace/CDC Stanley, AAC Innova/AA Proclaim, and AAC Cameron/P2711 populations where GEI accounted for 5.2–7.7% of the total variation, the M3 model showed 0.5–17.4% smaller prediction accuracies at CV1 than the main effect M2 model. Such results agree with previous studies that reported inconsistent results on the incorporation of GEI in the prediction models, with some studies reporting greater accuracies [15,25,31,43,44,45] and others reporting no advantage [46]. Overall, our results together with others demonstrate the difficulty in generalizing the integration of GEI in the prediction models, which should be considered on a population basis.

In the CV1 scenario, the overall average prediction accuracy of the M3 model across all environments in the AAC Innova/AAC Proclaim DH population was 1.9% greater than the diversity panel and 40.4–67.5% greater than all populations (Table S1C). In both CV2 and CV0 scenarios, the AAC Innova/AAC Proclaim population showed 18.8–20.5% greater accuracies over the AAC Cameron/P2711, 29.6–35.7% greater accuracies over both the Attila/CDC Go and the Peace/Carberry, 8.0–9.3% over the diversity panel, and 2.4–2.5% over the Peace/CDC Stanley population. Several studies have reported highly variable prediction accuracies depending on the genetic relationship between the training and prediction sets (population structure), trait heritability, population size, marker density, prediction models, the incorporation of GEI in the prediction models, and cross-validations scenarios [25,47,48,49,50,51,52]. In the present study, we did not observe any clear population structure within and among populations (Figure S1), but there were wider differences in broad-sense heritability (0.62–0.94), population sizes (153–208), and marker densities (1058–23,795), which may have contributed to the observed differences in the overall accuracies. The AAC Innova/AAC Proclaim population, for example, had the highest broad-sense heritability (0.94), followed by the diversity panel (0.89), with Attila/CDC Go showing the smallest heritability (0.62). Population size was the lowest in the Peace/CDC Stanley (153 RILs) and the highest in the Peace/Carberry (208 RILs), while marker density was the lowest in the Peace/CDC Stanley (1058 SNPs) and the highest in the diversity panel (23,795 SNPs). However, marker density seems not to have a clear effect on the prediction accuracies because the diversity panel that had three-fold more markers as compared with the AAC Innova/AAC Proclaim population (8066 SNPs) still showed 1.9–9.3% smaller accuracies depending on the CV scenarios.

## 4. Materials and Methods

### 4.1. Phenotyping and Genotyping

The present study was conducted on six Canadian spring wheat populations (Table S4) that consisted of an association mapping (diversity) panel of 196 historical and modern spring wheat varieties and unregistered lines [53], 208 RILs derived from the cross Peace/Carberry [54], 167 RILs derived from Attila/CDC Go [55], 153 RILs from Peace/CDC Stanley [56], 190 RILs from AAC Cameron/P2711 [57], and 190 DH lines from AAC Innova/AAC Proclaim [58]. The RILs were advanced to F_6_ using the single seed descent method, while the DH lines were developed from F_1_s at the Agriculture and Agri-Food Canada (AAFC) Research and Development Center in Lethbridge, AB, CA, using the wheat–maize hybridization method [59]. The diversity panel, the Peace/Carberry, and the Attila/CDC Go populations were previously used to compare prediction accuracies of 7 agronomic and end-use quality traits [36] and resistance to wheat diseases [22]. The methodologies for disease phenotyping, DNA preparations, genotyping, and genotype data filtering were described in our previous study [22]. Table S1C summarizes details of the phenotyping sites, years, and genotype information for each population. Briefly, the diversity panel and Peace/Carberry RIL populations were evaluated at 8 environments (site × year combinations) near Creston in British Columbia, the Lethbridge Research and Development Centre (Lethbridge RDC) in Alberta, and the University of Alberta South Campus in Edmonton, Alberta (Table S4C). The Attila/CDC Go and Peace/CDC Stanley RIL populations were evaluated in 3 environments near Creston, Lethbridge RDC, and Edmonton. The Peace/CDC Stanley RIL population was evaluated at three environments near Creston in 2016, at the University of Alberta South Campus Research Station in 2016, and at the Lethbridge RDC in 2017 in disease-screening field nurseries using a randomized incomplete block design with two replications. The above four populations, the parents of the RILs, resistant checks (Lillian and Carberry), and susceptible checks (AC Barrie, AC Crystal, and Park) were evaluated for reaction to stripe rust using either natural infection at Creston or artificial infection at the other sites, as described in our previous study [22]. Stripe rust severity was recorded when the susceptible checks displayed many pustules/lesions on the leaf surface and the resistant checks showed either no or a few pustules (lesions). Disease severity ratings were recorded either on a 0–9 scale or 0–100%, as follows: 0 = no infection, 1 = ≤10%, 2 = 11–20%, 3 = 21–30%, 4 = 31–40, 5 = 41–50%, 6 = 51%–60%, 7 = 61%–70%, 8 = 71%–80%, and 9 = ≥81% of the leaf area covered by pustules or lesions [60] when the susceptible checks displayed many pustules/lesions and the resistant checks had few or no pustules/lesions. We used the 0–9 scale in all statistical analyses.

The AAC Innova/AAC Proclaim DH population, the two parents, and checks were evaluated at five environments using a randomized complete block design with two replications both near Creston and the Lethbridge RDC in 2016 and 2020, and without replication at the Lethbridge RDC in 2017 [58]. The AAC Cameron/P2711 RIL population, the two parents, and checks were evaluated at five environments using a randomized complete block design with two replications both near Creston and the Lethbridge RDC in 2020, and without replication both at the Lethbridge RDC in 2018 and Crescent in 2018 and 2019 [57]. In both the AAC Innova/AAC Proclaim and AAC Cameron/P2711 populations, each line was planted in a 1-m-long row with a spacing of 25 cm between plants. AC Barrie, AC Crystal, and Morocco were used as susceptible checks, which were planted as border rows for disease spreading. The inoculum preparations, spray applications, and stripe rust severity scorings were done as described in a previous study [22].

The diversity panel, Attila/CDC Go, and Peace/CDC Stanley populations were genotyped with the Wheat 90 K iSelect single nucleotide polymorphisms (SNPs) arrays at the University of Saskatchewan Wheat Genomics lab, Saskatoon, Canada, as described in the previous studies [22,56]. The Peace/Carberry population was genotyped with a total of 36,626 markers (22,741 SilicoDArT markers with present/absent variation and 13,885 SNPs) using the DArTseq technology (https://www.diversityarrays.com (accessed on 28 June 2022)), Canberra, Australia. Both the AAC Innova/AAC Proclaim and AAC Cameron/P2711 populations were genotyped with the Wheat 90 K iSelect SNPs array using the Agriculture and Agri-Food Canada’s (AAFC) facility at the Modern Research and Development Center [57,58]. In the diversity panel, we retained SNPs with minor allele frequencies of > 5% and a missing data point of <20%. In the remaining five biparental populations, we retained all markers that had <20% missing data, were polymorphic between their respective parents, and had a segregation distortion of *p* > 0.01. The final number of molecular markers retained for genome-wide prediction varied from 1058 in the Peace/CDC Stanley to 23,793 in the diversity panel (Table S5).

### 4.2. Statistical Analyses

The first three PCs from principal component analysis (PCA) were obtained in TASSEL v5.2.82 [61], which were plotted to understand population structure within and among populations in CurlyWhirly v1.21.08.16 (https://ics.hutton.ac.uk/curlywhirly (accessed on 28 June 2022). The best linear unbiased estimators (BLUE), variance component analyses, broad-sense heritability, and prediction accuracies were computed as described in our previous study [22]. Briefly, genome-wide prediction accuracies were computed using the phenotypic model (M1), the main effects of phenotype and marker (M2), and the M3 model that incorporated GEI. The M1 model attempts to explain the response of the *j*^th^ wheat line in the *i*^th^ environment ($y_{ij}$) by accounting for the effects of the environment ($E_{i}$), the line ($L_{j},$) and residual variance ($\varepsilon_{ij}$) as $y_{ij}=\mu +E_{i}+L_{j}+e_{ij}$, where μ is an intercept. The M2 model extends the M1 model by adding the the main effect of molecular markers data ($g_{j}$) as $y_{ij}=\mu +E_{i}+L_{j}+g_{j}+e_{ij}$. The M3 model extends the M2 model by incorporating GEI effects ($gE_{ij}$) as $y_{ij}=\mu +E_{i}+L_{j}+g_{j}+gE_{ij}+e_{ij}.$ The M1 model does not allow borrowing of information among lines, whereas both the M2 and M3 model allow borrowing of information among the lines to predict performance of lines in environments where the lines were not observed.

Each model was assessed to mimic (i) predicting the performance of a subset of newly developed wheat lines that have not been tested in any environment (CV1), (ii) sparse testing where a subset of lines was evaluated in some environments but not in others (CV2), and (iii) predicting a future environment where all lines were tested in some environments but not in other target environments (CV0). The analyses in both CV1 and CV2 were performed by randomly assigning 25% of the wheat lines in each population into a testing (prediction) set and the remaining 75% of the lines into a training (calibration) set in R and the Bayesian Generalized Linear Regression (BGLR) package [62]. Predictions in the CV0 scenario were made only once, using a leave-one-out method with no random process involved to assign lines into folds. All prediction analyses were done by setting the number of replicates of the random cross-validation to 40, the number of iterations to 12,000, burn-in to 2000, and thinning to 5. The Pearson’s correlation between the observed phenotypes and genomic estimated breeding values (GEBVs) in the testing set was used as a measure of prediction accuracy. The overall changes in prediction accuracies between pairs of models were computed as $\Delta \mathrm{PA}=100\times \frac{\left(M2-M1\right)}{M2}$ or $100\times \frac{M3-M1}{M3}\;\mathrm{or}\;100\times \frac{M3-M2}{M3}$. Tests for statistical differences in prediction accuracies among models, CV scenarios, populations, and environments were done using analysis of variance (ANOVA) and the Tukey–Kramer multiple comparisons of means implemented in JMP statistical discovery software [63] v16.0.0. Correlations analysis, regression analysis, bar graphs, and box plots were also generated using JMP v16.0.0.

## 5. Conclusions

The predictive ability of the CV1 scenario was significantly smaller than both the CV2 and CV0 scenarios regardless of the models and genetic backgrounds, suggesting the challenge in successfully implementing GS to develop stripe-rust-resistant spring wheat without any phenotype data in the testing (prediction) set. The high prediction accuracies observed in both the CV2 and CV0 scenarios regardless of the models and populations, on the other hand, suggest a great potential to use GS in stripe rust resistance breeding by phenotyping either a subset of lines (sparse testing) or all lines in a few environments and then predicting future environments using either the M2 or M3 models. The incorporation of GEI in the M3 model showed significant differences in prediction accuracies in the CV1 but not in the CV2 or CV0 scenario. The M3 model that incorporated GEI in the CV1 scenario showed an improvement in prediction accuracies in half of the populations but a reduction in accuracies in the remaining half of the populations. Overall, the effect of incorporating GEI in the M3 model differed depending on the genetic backgrounds and cross-validations scenarios, which suggests the need to look at individual populations. To the best of our knowledge, this is the first comprehensive study that investigated the effect of genotype by environment interaction on the predictive ability of stripe rust resistance across diverse types of spring wheat populations that involved historical and modern varieties, RILs, and DH lines. The results provide a broader perspective of genome-wide prediction accuracies for further stripe rust resistance breeding in wheat.

## Figures and Tables

**Table 1:** Summary statistics of variance component analyses and broad-sense heritability of stripe rust in six spring wheat populations evaluated at three to eight field environments.

Statistic	Diversity Panel	Peace/Carberry	Attila/CDC Go	Peace/CDC Stanley	AAC Innova/AAC Proclaim	AAC Cameron/P2711
Genotype variance (σ^2^g)	3.00	0.53	1.28	4.55	4.80	3.65
Environment variance (σ^2^e)	1.15	0.83	0.99	2.28	0.04	1.18
G × E interaction (σ^2^ge)	1.19	0.36	0.84	1.73	0.71	1.65
Residual (error) variance	1.54	0.96	1.24	1.12	1.58	1.78
Grand mean	3.76	2.07	3.85	3.66	2.83	3.59
Least significant difference	1.35	0.83	1.68	2.29	1.56	2.03
Mean number of replicates	2.00	2.00	2.33	2.00	1.80	1.40
No. of environments	8.00	8.00	3.00	3.00	5.00	5.00
*p* value for genotypes	0.01	0.01	0.01	0.01	0.01	0.01
*p* value for environments	0.01	0.01	0.25	0.01	0.51	0.01
*p* value for G × E interaction	0.01	0.01	0.01	0.01	0.01	0.01
Broad-sense heritability	0.89	0.72	0.62	0.75	0.94	0.82

**Table 2:** The minimum (min), maximum (max), mean, and standard deviation (Std) of stripe rust prediction accuracies for six spring wheat populations based on three models (M1, M2, and M3) and three random cross-validations scenarios (CV1, CV2, and CV0). Each value was based on 40 iterations. See Table S1 for details on prediction accuracies.

		CV1				CV2				CV0			
Population	Model	Min	Max	Mean	Std	Min	Max	Mean	Std	Min	Max	Mean	Std
AAC Cameron/P2711	M1	−0.12	−0.09	−0.10	0.01	0.55	0.75	0.65	0.08	0.57	0.79	0.68	0.08
	M2	0.19	0.30	0.24	0.04	0.55	0.76	0.66	0.08	0.57	0.80	0.69	0.08
	M3	0.14	0.30	0.24	0.06	0.53	0.76	0.66	0.09	0.57	0.80	0.68	0.08
AAC Innova/AAC Proclaim	M1	−0.09	−0.06	−0.07	0.01	0.77	0.87	0.82	0.04	0.78	0.89	0.84	0.04
	M2	0.42	0.55	0.49	0.05	0.76	0.87	0.82	0.04	0.78	0.89	0.84	0.04
	M3	0.40	0.57	0.48	0.07	0.76	0.87	0.83	0.04	0.78	0.89	0.84	0.04
Attila/CDC Go	M1	−0.12	−0.10	−0.11	0.01	0.49	0.59	0.53	0.06	0.51	0.61	0.54	0.06
	M2	0.22	0.29	0.25	0.04	0.50	0.60	0.54	0.05	0.50	0.58	0.53	0.04
	M3	0.23	0.33	0.29	0.05	0.51	0.64	0.57	0.07	0.51	0.6	0.54	0.05
Diversity panel	M1	−0.10	−0.06	−0.08	0.01	0.64	0.83	0.75	0.07	0.66	0.85	0.76	0.07
	M2	0.32	0.53	0.45	0.08	0.63	0.84	0.75	0.07	0.65	0.86	0.76	0.08
	M3	0.33	0.57	0.47	0.08	0.67	0.84	0.76	0.07	0.65	0.86	0.76	0.08
Peace/Carberry	M1	−0.11	−0.08	−0.09	0.01	0.40	0.69	0.57	0.11	0.42	0.72	0.59	0.12
	M2	0.15	0.30	0.22	0.05	0.4	0.69	0.57	0.11	0.42	0.72	0.59	0.11
	M3	0.14	0.32	0.22	0.09	0.38	0.69	0.57	0.11	0.42	0.72	0.59	0.12
Peace/CDC Stanley	M1	−0.10	−0.07	−0.09	0.01	0.67	0.91	0.82	0.13	0.68	0.91	0.82	0.13
	M2	0.20	0.24	0.22	0.02	0.67	0.91	0.82	0.13	0.68	0.91	0.82	0.13
	M3	0.18	0.21	0.19	0.06	0.67	0.90	0.80	0.12	0.68	0.91	0.82	0.13

## Data Availability

All relevant results are included within this article and its supplementary files. The original phenotype and genotype data are available on request from the corresponding author.

## Supplementary Materials

The following are available online at https://www.mdpi.com/article/10.3390/plants11131736/s1, Figure S1. Plots of PC1 (that accounted for 4.6–11.3% of the genetic variation depending on the population), PC2 (4.2–7.6%), and PC3 (3.7–6.6%) from principal component analysis to show population structure of the six spring wheat populations based on 1058 to 23795 polymorphic markers. See Table S4C for the number of markers used in each population; Table S1. Stripe rust prediction accuracies of six populations based on three models (M1, M2, and M3) and three random cross-validation scenarios (CV1, CV2, and CV0). In both CV1 and CV2, each environment is an average of 40 iterations, whereas CV0 is represented just by one with no randomization; Table S2. Analysis of variance (ANOVA) with Tukey–Kramer HSD pairwise test to compare prediction accuracies among three models (M1, M2, and M3) and cross-validations (CV1, CV2, and CV0) scenarios in six populations. For details, see Supplementary Table S1. In the Tukey–Kramer column, means sharing the same letters are not significantly different at *p* < 0.05; Table S3 Analysis of variance (ANOVA) with Tukey–Kramer HSD pairwise comparisons of means to compare the predictive ability of the M3 model with three cross-validations (CV1, CV2, and CV0) scenarios in six populations. For details, see Supplementary Table S1. In the Tukey–Kramer column, means sharing the same letters are not significantly different at *p* < 0.05; Table S4. Summary of the six spring wheat populations used in the present study; Table S5. Summary of the number and chromosomal distribution of the polymorphic markers used in the present study.
