# Supplementary material for: Genomic Prediction Accuracy of Stripe Rust in Six Spring Wheat Populations by Modeling Genotype by Environment Interaction
Source: Plants (Basel). 2022 Jun 30;11(13):1736. doi: 10.3390/plants11131736 (PMC9269065; doi:10.3390/plants11131736)
Supplement: Supplementary file 1 [file plants-11-01736-s001.zip › Figure S1 Population structure R1.pptx]

## Slide 1
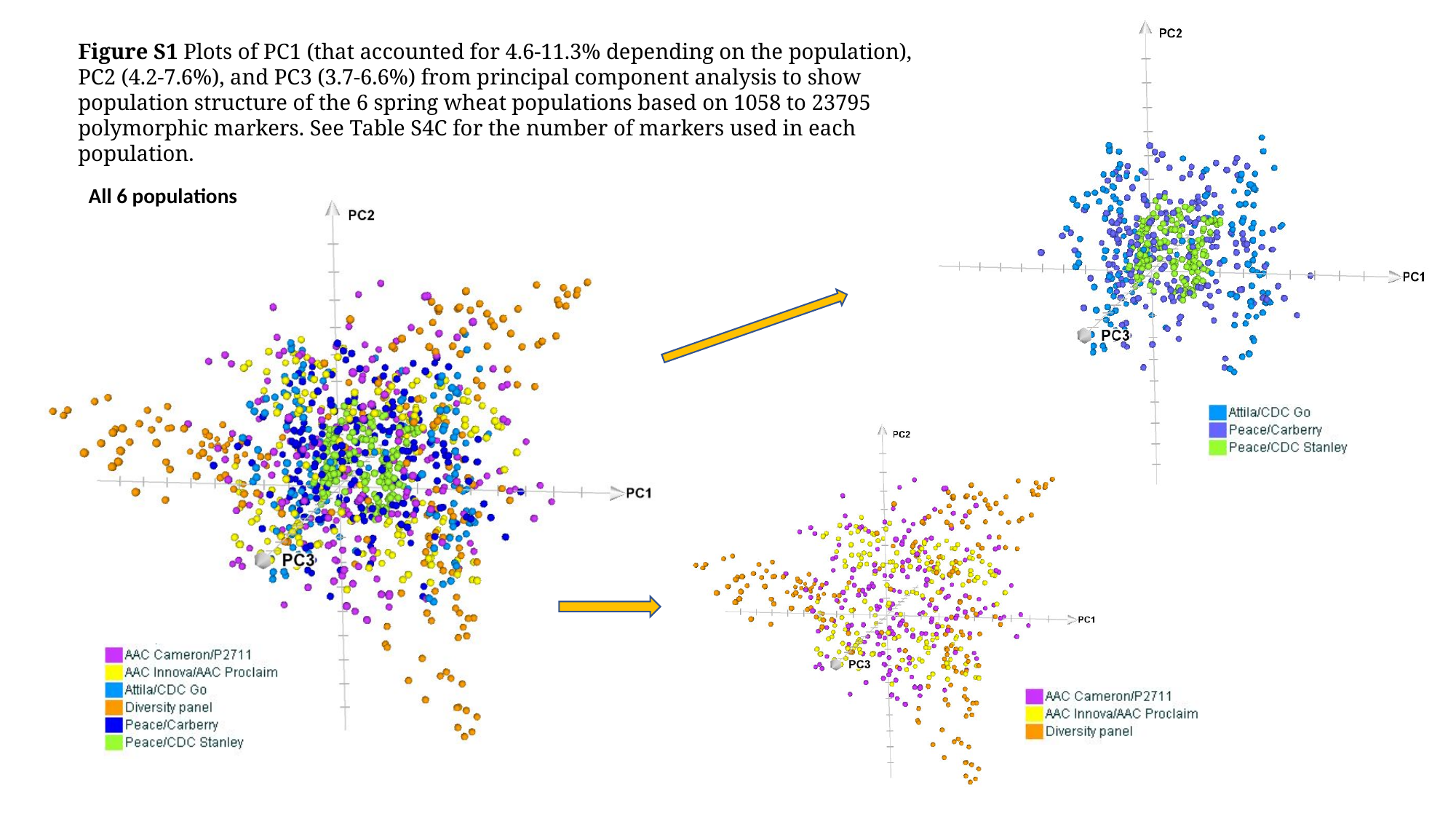

Figure S1 Plots of PC1 (that accounted for 4.6-11.3% depending on the population), PC2 (4.2-7.6%), and PC3 (3.7-6.6%) from principal component analysis to show population structure of the 6 spring wheat populations based on 1058 to 23795 polymorphic markers. See Table S4C for the number of markers used in each population.
All 6 populations
